# Doppler ultrasonographic evaluation of brachial and femoral veins, and coagulation and lipid profiles in dogs following open splenectomy

**DOI:** 10.1038/s41598-019-51924-0

**Published:** 2019-10-25

**Authors:** Hussein Awad Hussein, Ahmed Ibrahim, Marwa F. Ali, Ahmed F. Ahmed

**Affiliations:** 1 Internal Veterinary Medicine, Department of Animal Medicine, Faculty of Veterinary Medicine, Assiut University, Assiut, 71526 Egypt; 2 Assistant Consultant of Surgery, Anesthesiology and Radiology, Veterinary Teaching Hospital, Faculty of Veterinary Medicine, Assiut University, Assiut, 71526 Egypt; 3 Department of Veterinary Pathology and Clinical Pathology, Faculty of Veterinary Medicine, Assiut University, Assiut, 71526 Egypt; 4 Department of Surgery, Anesthesiology and Radiology, Director of Veterinary Teaching Hospital, Faculty of Veterinary Medicine, Assiut University, Assiut, 71526 Egypt

**Keywords:** Ultrasound, Diseases

## Abstract

In dogs, splenectomy is mandatory as an emergency following splenic rupture with resultant hemoperitoneum and hypotensive shock. The present work aimed to evaluate the Doppler ultrasonographic parameters of brachial and femoral veins in splenectomized dogs and to investigate the effect of splenectomy on the coagulation and lipid profiles. A total number of 9 dogs underwent clinical, abdominal ultrasonographic and laboratory examinations prior to the surgical operation and kept for 60-day observation period post-splenectomy. Follow-up ultrasonography revealed no serious complications post-splenectomy. Both brachial and femoral veins were imaged medial to their corresponding arteries. Doppler ultrasonographic parameters of both veins showed no significant changes throughout the study period (P > 0.05). Haematological analysis revealed development of anemia, leukocytosis, and thrombocytosis in dogs post-splenectomy. Coagulation profile exhibited no significant variations in prothrombin and activated partial thromboplastin times (P > 0.05). In comparison with their baseline values, the mean concentrations of total cholesterol, low-density lipoprotein, and triglycerides were significantly increased 30-day post-splenectomy. In conclusion, it may seem that open splenectomy has no influence on the Doppler ultrasonographic indices of brachial and femoral veins with no evidence of deep vein thrombosis in dogs. However, persistent leukocytosis and thrombocytosis, as well as altered lipid profile may increase the risk of vascular complications with the long run. Therefore, a further long-term study may be required.

## Introduction

Spleen is a part of the reticuloendothelial systems and has many functions. Spleen consists of white and red pulps. The white pulp represents the main lymphoid centers within the spleen and involved in the production of B and T lymphocytes, while the red pulp is responsible for erythrocyte and platelet storage, and extramedullary hematopoiesis^1^. Spleen has a phagocytic function represented by the filtration process resulting from moving of the blood slowly through the splenic sinusoids in the red pulp lined with macrophages actively ingesting materials, which does not easily pass around them^2^. The Absence of this extremely sensitive filter may permit particulate matter and damaged cells to persist in the bloodstream, therefore enhancing and activating the vascular endothelium resulting in a shift in vascular homeostasis toward increased coagulation^3^. In addition, spleen is necessary for lipid metabolism^4^.

Splenic rupture, usually rooted from splenic hemangiosarcoma, is a common cause of intra-abdominal hemorrhage in dogs^5^. Therefore, most of the splenic affections have been cured by splenectomy. In dogs, splenectomy is indicated for splenic tumors, torsion, and traumatization^6^. Various complications may develop after splenectomy including post-surgical infection, vascular complications and pulmonary hypertension^2^.

Doppler ultrasonography, because of its accessibility, noninvasiveness, safety and easy for the patient, has become the method of choice in the diagnosis of vascular disease in human medicine^7^. Furthermore, Doppler ultrasonography has brought a revolution in treatment and monitoring of diseases of the blood vessels. In addition, it allows simultaneous imaging and spectral analysis of vascular flow patterns^8^. These patterns have the potential to provide both qualitative and quantitative functional information relative to a particular vascular disease^9^.

Several studies have investigated the hematologic changes associated with splenectomy in dogs^10^, goat^11^ and human^12^. From the clinical point of view, the available information regarding the possible consequences of splenectomy in the animals has not been well characterized and most of the data in this regard has originated from human sources. In human medicine, deep vein thrombosis (DVT) and thromboembolism may develop as a vascular complication accompanied splenectomy^2^. To the best of the authors’ knowledge, there are no literature evaluating the risk of vascular thrombosis or evaluating the consequent coagulation and lipid profiles associated with open splenectomy in dogs. Therefore, the objectives of this study were as follows: (1) evaluate the Doppler ultrasonographic indices, including mean blood velocity, blood flow rate and congestion index, of brachial and femoral veins in splenectomized dogs; (2) assess the possible variations of the coagulation and lipid profiles post-splenectomy.

## Materials and Methods

### Animals and study design

The present work was ethically approved by the Animal Care and Welfare Committee of Faculty of Veterinary Medicine, Assiut University, Assiut, Egypt. All institutional ethical regulations were followed during study procedures. The current research was carried out as a prospective cohort study on 9 adult clinically healthy dogs of beagle breed, of both sexes (4 males, and 5 non-lactating and non-pregnant females). The mean ± standard error (SE) of age was 4.3 ± 0.05 years, and weight was 15 ± 0.8 kg. Before carrying out the study, all dogs were examined clinically and ultrasonographically, as well as they subjected for laboratory examinations. Dogs identified to have any indication of splenic diseases or abnormal laboratory findings were excluded from the study. All animals were housed indoors in individually numbered cages with balanced diets and water *ad libtum*. All dogs were regularly monitored for any illness throughout the study period (60 days). At the end of the study, the dogs were euthanized by intravenous (IV) injection of an over dosage of sodium thiopental (200 mg/kg)^13^, and then the animal remains were handled appropriately and in accord with state and local law, and then used for teaching purposes.

### Surgical procedures

Dogs fasted for 12 hours (h) for food and 3 h for water before the surgery. For anesthesia, intravenous (IV) 2 mg/kg ketamine 5% (Ketamine, Sigma-tec Pharmaceutical Industries, SAE, Egypt) and 1 mg/kg xylazine 2% (Xyla-Ject, ADWIA Co., SAE, Egypt), in one syringe were used for induction, and then maintained by total intravenous anesthesia (TIVA) using 10 mg/kg/h ketamine 5% and 1 mg/kg/h xylazine 2%.

**Figure 1:**
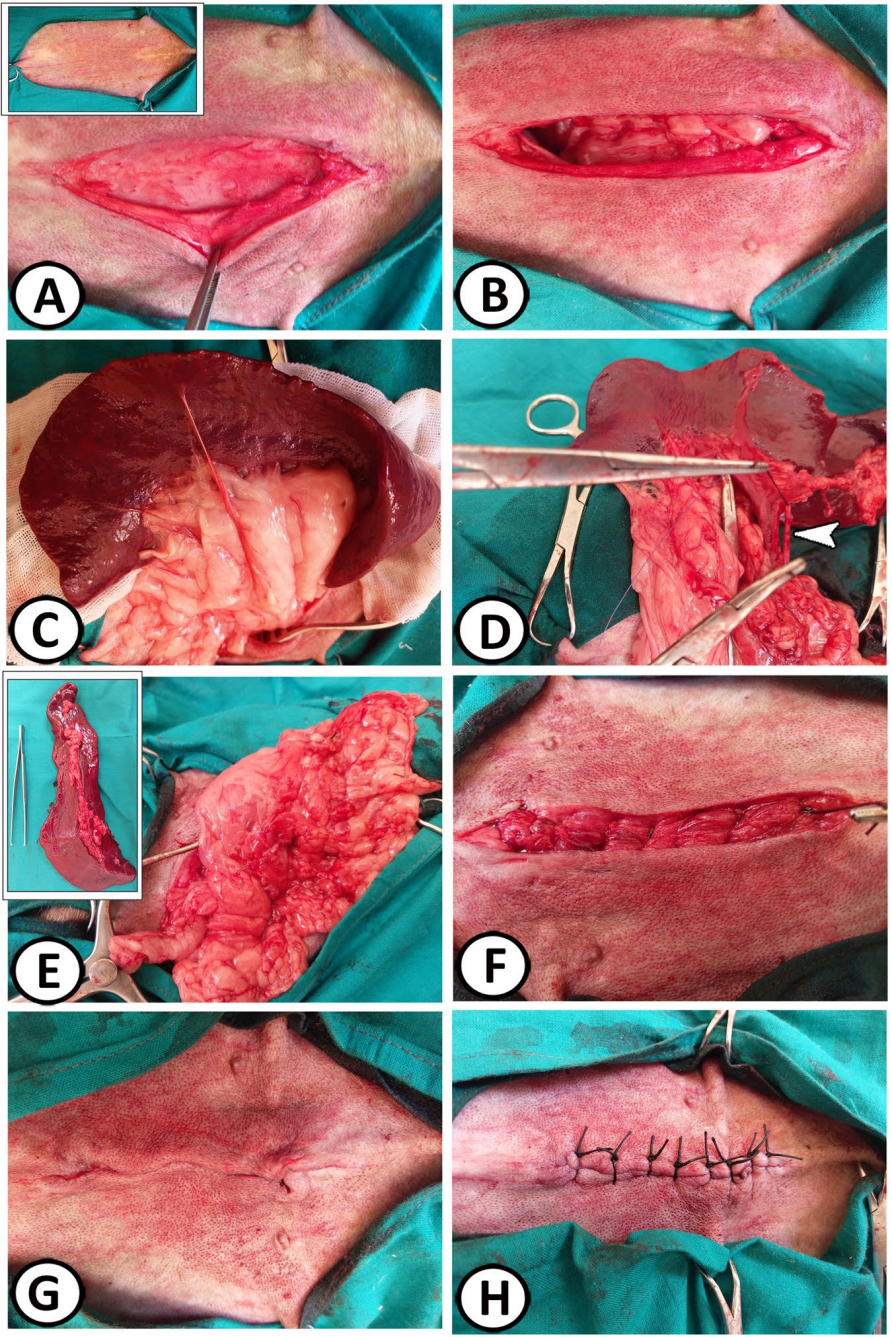
Surgical procedures of open splenectomy. (**A**) Initial skin incision was made with dissection of subcutis and exposure of the linea alba. The inserted box pointed to the aseptic preparation of the operation field. (**B**) Midline celiotomy. (**C**) Exteriorization of the spleen with gastrosplenic ligament and vessels. (**D**) The site of cutting of the splenic blood vessels between the two ligatures (arrowhead). (**E**) The gastrosplenic ligament after complete excision of the spleen. The inserted box showed the excised spleen. (from **F**–**H**) Closure of the abdominal wall.

The dog was positioned on the surgical table in a dorsal recumbency. The entire abdominal region was surgically prepared by clipping, shaving, and repeated scrubbing with 10% povidone-iodine solution. The whole animal was draped except for the operative site. The spleen was approached via a ventral midline abdominal incision through the skin, subcutaneous tissue, and linea alba; extending from the xyphoid to a point caudal to the umbilicus (Fig. 1). The spleen was exteriorized and isolated by wet abdominal sponges.

Each individual splenic vessel was double ligated using synthetic absorbable suture material (Vicryl, 2-0) and transected as near as possible from the splenic hilus, starting at the tail of the spleen. The short gastric branches as well as pancreatic branch were preserved during surgery. Any attached mesentery was ligated before being transected. The spleen was removed from the surgical area. The ligated splenic blood vessels as well as the whole abdomen were checked for any hemorrhage. The abdominal incision was closed in three layers. The *linea alba* and subcutaneous tissue were closed individually, with Maxon 0, Maxon), in a simple continuous suture pattern, while the skin was closed with silk sutures, 0 in an interrupted suture pattern^14,15^. The duration of surgery was ranged from 35 to 40 min. Wound dressing was performed daily using 10% povidone-iodine solution and the incisional sites were evaluated for infection, inflammation, or dehiscence daily until removing the skin stitches. Skin sutures were removed 10 days (seven dogs) and 12 days (two dogs) post-operation. The incisional sites were cleaned and healed completely by the first intention without any complications.

Ketoprofen 2 mg/kg (Ketofan, 2 ml ampoule containing 100 mg ketoprofen, Amriya Pharm. Ind., Alexandria- Egypt), was administered intramuscular (IM) immediately after surgery and then once daily for three successive days post-operatively. All dogs were administered cephalexin (cephalexin monohydrate, 1000 mg ampoules, Rameda Pharmaceuticals, SAE, Egypt) at a dose of 25 mg/kg intramuscular (IM), once daily for five successive days post-operatively (PO).

### Doppler ultrasonographic examination

A triplex scan ultrasonographic device (MyLabTMOne VET, Esaote, Italy) was used. It had two multi-frequency transducers, micro convex from 3.3 to 5 MHz and linear from 6 to 10 MHz. For preparation of ultrasonographic examination, all animals were placed in lateral recumbency, and the medial skin surfaces of arm and thigh were clipped, shaved, and disinfected with 70% alcohol.

**Figure 2:**
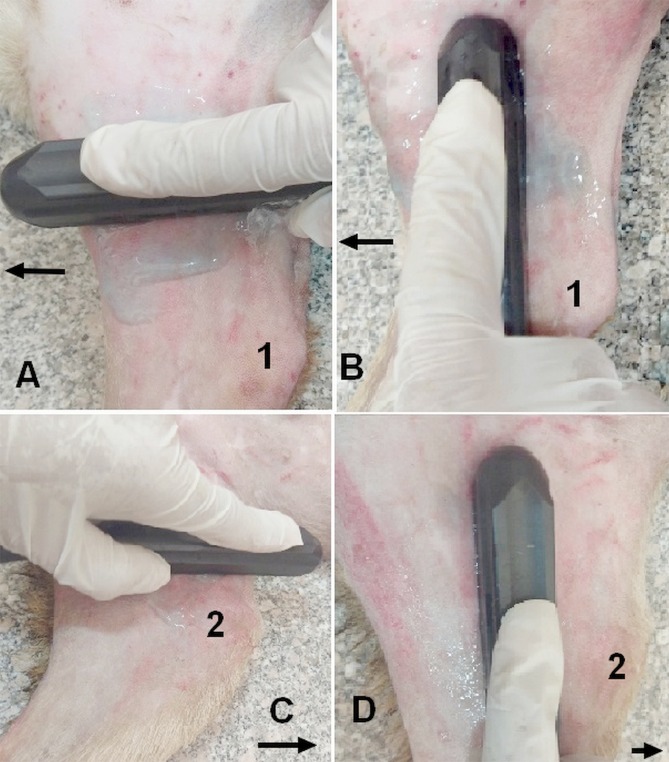
Ultrasound probe positions. (**A**) Firstly the probe was placed in a transverse plane for the arm to allocate the brachial vein and calculation its diameter, then (**B**) the probe position was redirected to be in a longuitidinal plane for other ultrasonographic measurments. (**C**) In a transverse plane above the stiffle joint, the femoral vein was identified, then (**D**) the probe was turned to be parrallel for the vein of interest for measurments of Doppler indices. 1 = elbow joint; 2 = stiffle joint. The arrows refer to the direction of the head of dog.

**Figure 3:**
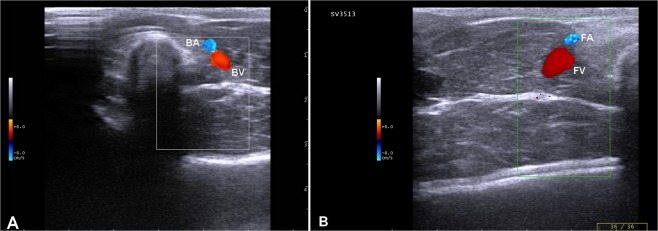
Color Doppler ultrasonographic scan for brachial (**A**) and femoral (**B**) veins. Note both veins are located medial for their corresponding arteries. BA = brachial artery; BV = brachial vein; FA = femoral artery; FV = femoral vein.

Default adjustments of the ultrasound device for the small parts and the resolution frequency were used for all cases. B-mode and color flow imaging were done initially to identify the veins of interest, where the ultrasound probe was positioned in a transverse and longitudinal plane (Fig. 2). To access the brachial vein, the area extending from the axilla to the medial aspect of the elbow joint was scanned and the vein was identified at the mid-way of the arm and used as a landmark. The brachial vein was closely related to the brachial artery (Fig. 3A). The brachial veins were differentiated from the artery by its blood flow direction and spectral pattern. The area extending from the inner aspect of the groin to the stifle joint was examined and the femoral vein was identified by lack of pulsatility, by compressibility with minimal pressure and respiratory phasicity. In the transverse plane, the femoral vein was identified directly medial to the femoral artery (Fig. 3B).

Before Doppler ultrasonographic evaluation, the diameters of brachial and femoral veins were measured then vessel cross-sectional areas were calculated using the following formula^16^:

$$A=({D}^{2}\times \pi )\div4$$

where *A*: vein area; *D*: vein diameter; *π*: 3.14.

In sagittal views, color and spectral Doppler examination was carried out for the brachial and femoral veins to evaluate blood flow direction and velocity within the vessel. Firstly the vein was located in a transverse plane then the probe was slightly rotated to obtain a longitudinal image for the vessel. The angle between the sound waves and the flow direction in all evaluated vessels was kept less than 60 degrees in all scans. The cursor was introduced toward the vein and the sample size (gate) was set to be 2 mm for brachial and femoral veins. In all cases, Doppler waveforms were recorded at a pulse repetition frequency (PRF) that was sufficient to prevent aliasing artifact. The mean blood flow velocity (*Vmean*) was assessed through the uniform insonation technique by the ultrasound device; angle correction was used in all cases to calculate the velocities accurately. Doppler ultrasonographic examinations were conducted for all animals every 15 days from 0 (pre-splenectomy) to 60 days post-splenectomy.

Based on the obtained data, the blood flow rate (BFR) and the congestion index (CI) were assessed. To calculate BFR of the examined vein, the following formula was used^8^:

$$BFR(ml/\min )=Vmean\,(cm/{\min })\times A(cm{)}^{2}\times 60$$

where *Vmean*: the mean blood flow velocity of the examined vein; A: the vein area.

To calculate CI of the examined vein, the following formula was used^17^:

$$CI(cm.sec)=A(cm{)}^{2}{\div}Vmean\,(cm/sec)$$

### Blood sampling and laboratory analysis

Blood samples with ethylene diamine tetraacetic acid (EDTA) for blood picture, with sodium citrate for hemostatic profile and without anticoagulant for serum biochemical analysis were collected from cephalic vein. For blood picture and hemostatic parameters, 9 blood samples were obtained from each animal as 0 (pre-splenectomy), and 3 h, and 1, 4, 8, 15, 30, 45- and 60-days post-splenectomy. For lipid and other biochemical indices, 6 blood samples were drawn from each dog as 0 (pre-splenectomy), 5, 15, 30, 45, and 60 days post-splenectomy.

Hematological analyses including total red blood cells (RBCs) count, hemoglobin (Hb) concentration, hematocrit (HCT), total white blood cells (WBCs) count, and platelets (PLT) count were automatically measured (Exigo veterinary hematology analyzer, Sweden). After centrifugation of the sodium citrated tubes, the plasma samples were harvested and used immediately for estimation of prothrombin time (PT) and activated partial thromboplastin time (APTT) using commercial test kits (BIO MED DIAGNOSTICS, Germany). After centrifugation of the plain tubes, serum samples were collected and then frozen at −20 °C for 1 week; subsequently, analysis of biochemical parameters including total cholesterol, high-density lipoproteins (HDL), low-density lipoproteins (LDL), triglycerides (TG), alanine aminotransferase (ALT), aspartate aminotransferase (AST), urea, and creatinine were performed using commercial test kits (Spectrum Diagnostics, Egypt).

### Statistical analysis

Data were expressed as mean ± SE (standard error) and were analyzed statistically using SPSS software (IBM SPSS analytical program for Windows Version 21; SPSS GmbH, Munich, Germany). The normal distribution of all parameters was tested using Kolmogorov-Smirnov normality test. All parameters were normally distributed. To estimate the significant changes in different variables pre-splenectomy and post-splenectomy, the paired-sample *t*-test was carried out. Results were considered significant at P < 0.05.

### Ethics approval and consent to participate

The present study was approved by The National Ethical Committee of Faculty of Veterinary Medicine, Assiut University, Assiut, Egypt.

## Results

Follow-up clinical and abdominal ultrasonographic examinations revealed neither hemorrhage nor serious complications following open splenectomy.

### Doppler ultrasonographic imaging

**Figure 4:**
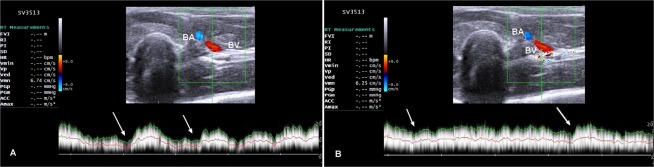
Triplex ultrasound scan for the brachial vein approximately at the middle of medial aspect of arm. (**A**) A longitudinal scan before splenectomy (0) with a cross sectional area of 2.5 cm^2^ and blood flow rate of 1011 ml/min. (**B**) A scan for the same brachial vein after 60 days following splenectomy with a cross sectional area of 2.8 cm^2^ and the blood flow rate was calculated as 1050 ml/min. note: the spectral waveforms show respiratory phasicity (arrows). BA = brachial artery; BV = brachial vein.

**Figure 5:**
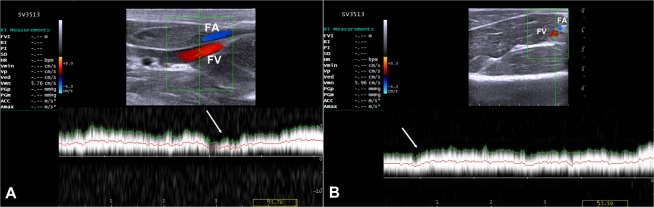
Spectral Doppler scan of the femoral vein approximately at the middle of medial side of thigh. (**A**) A longitudinal scan before splenectomy (0) with a cross sectional area of 4.3 cm^2^ and the blood flow rate was 1486 ml/min. (**B**) A scan for the same femoral vein after 60 days of surgical operation with a cross sectional area of 4.1 cm^2^ and the blood flow rate was 1466 ml/min. the spectral waveforms show respiratory phasicity (arrows). FA = femoral artery; FV = femoral vein.

Table 1 shows serial variations of the mean blood velocity, blood flow rate and congestion index of brachial and femoral veins before and after splenectomy. No significant changes were noticed throughout the study (*P* > 0.05). The wall of the brachial vein was thin and smooth with consistent color fill, indicating absence of color filling defects (Fig. 4). Triplex ultrasonographic evaluation of femoral vein revealed no evidence for the presence of flow disturbance and incompetence, indicating no venous reflux (Fig. 5). The blood flow in both veins was laminar. In addition, both veins were completely compressible with minimal pressure on the probe, indicating patency and no evidence for DVT. Furthermore, the spectral waveform analysis of both veins showed the respiratory phasicity, indicating fall venous flow with inspiration and rise with expiration (Figs 4 and 5). In addition, no pulsatility, aliasing, or vein deformity was imaged, as well as no collateral vessels were seen.

**Table 1:** Summary of Doppler ultrasonographic parameters of brachial and femoral veins in dogs (n = 9) pre- and post-splenectomy.

Parameters^*^	Time intervals before and after splenectomy (days)					*P*-value
	0	15	30	45	60	
**Brachial vein**						
Diameter (mm)	5.6 ± 0.2	5.8 ± 0.1	5.6 ± 0.2	5.4 ± 0.2	5.3 ± 0.3	0.642
Blood flow velocity (cm/sec)	6.5 ± 0.5	6.4 ± 0.5	6.5 ± 0.3	6.6 ± 0.4	6.3 ± 0.2	0.986
Blood flow rate (ml/min)	984 ± 112	1012 ± 88	951 ± 66	953 ± 112	858 ± 85	0.822
Congestion index (cm.sec)	0.39 ± 0.03	0.43 ± 0.04	0.38 ± 0.05	0.37 ± 0.03	0.36 ± 0.04	0.717
**Femoral vein**						
Diameter (mm)	7.2 ± 0.3	7.4 ± 0.1	7.1 ± 0.5	7.0 ± 0.4	6.9 ± 0.4	0.909
Blood flow velocity (cm/sec)	6.2 ± 0.4	6.1 ± 0.2	5.9 ± 0.5	6.0 ± 0.3	6.2 ± 0.3	0.985
Blood flow rate (ml/min)	1544 ± 198	1583 ± 70	1419 ± 194	1399 ± 143	1413 ± 168	0.889
Congestion index (cm.sec)	0.65 ± 0.03	0.70 ± 0.04	0.71 ± 0.1	0.67 ± 0.1	0.62 ± 0.2	0.886

*Data are mean ± standard error of the mean.

### Effect of open splenectomy on the blood picture

**Figure 6:**
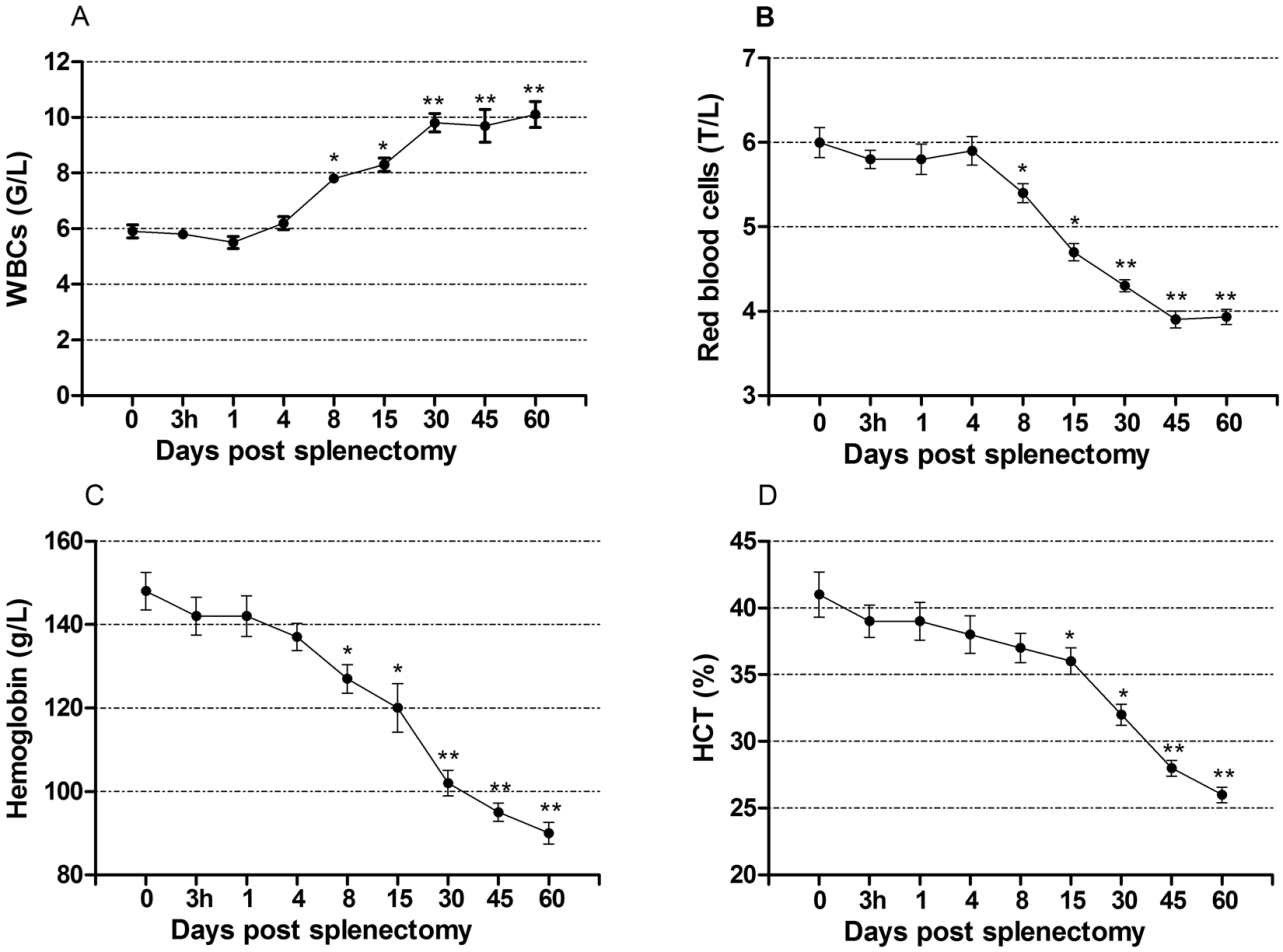
The values (mean ± SE) of hematological parameters in splenectomized dogs. (**A**) White blood cells count (WBCs). (**B**) Red blood cells count. (**C**) Hemoglobin concentration. (**D**) Hematocrit (HCT). * *P* < 0.05, ** *P* < 0.01.

The effect of splenectomy on the mean values of WBCs, RBCs, Hb and HCT is displayed in Fig. 6. At 8 days post-splenectomy, the level of WBCs was higher than the pre-splenectomy level (*P* < 0.05) and remained higher till the end of the study. The mean values of RBCs, Hb and HCT were significantly decreased 8 days after splenectomy (*P* < 0.05) and continued lower than the pre-splenectomy values till the end of study period (*P* < 0.01).

### Effect of open splenectomy on the coagulation profile

**Figure 7:**
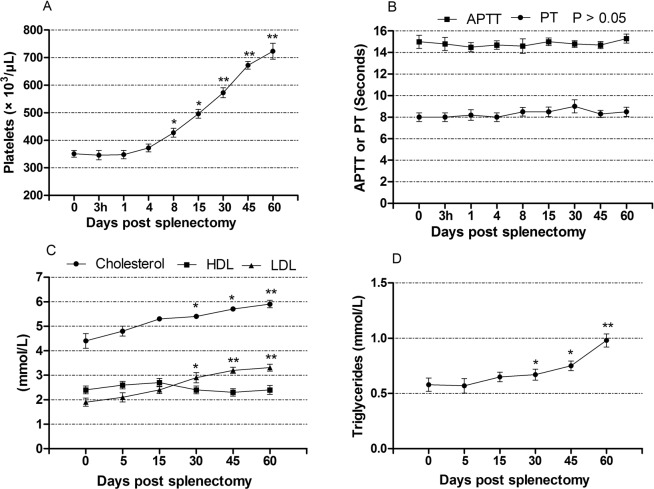
The values (mean ± SE) of coagulation and lipid profiles in dogs following splenectomy. (**A**) Platelets counts. (**B**) Prothrombin time (PT) and activated partial thromboplastin time (APTT). (**C**) Cholesterol, HDL and LDL. (**D**) Triglycerides. * *P* < 0.05, ** *P* < 0.01.

Figure 7 shows the effect of open splenectomy on the blood PLT level and coagulation times (PT and APTT). The mean PLT values increased significantly 8 days after splenectomy (*P* < 0.05). In comparison with pre-splenectomy levels, the highest value of PLT was seen 60 days post-splenectomy (351 ± 12 vs. 723 ± 29, *P* < 0.01). Coagulation times PT and APTT exhibited no significant changes during the study period (*P* > 0.05).

### Effect of open splenectomy on the lipid profile

The influence of open splenectomy on the concentrations of total cholesterol, HDL, LDL and TG is illustrated in Fig. 7. Serum concentrations of total cholesterol and LDL were significantly increased 30 days after splenectomy (*P* < 0.05). In comparison with their pre-splenectomy levels, the highest values of total cholesterol and LDL were observed 60 days post-splenectomy (4.4 ± 0.3 mmol/L vs. 5.9 ± 0.15 mmol/L, *P* < 0.01) and (1.9 ± 0.17 mmol/L vs. 3.31 ± 0.14 mmol/L, *P* < 0.01), respectively. In contrast, serum levels of HDL revealed non-significant variations (*P* > 0.05). Moreover, serum concentrations of TG were significantly increased post-splenectomy in comparison with pre-splenectomy values at day 30 (0.67 ± 0.05 mmol/L vs. 0.58 ± 0.06 mmol/L; *P* < 0.05), day 45 (0.75 ± 0.04 mmol/L vs. 0.58 ± 0.06 mmol/L; *P* < 0.05) and day 60 post-splenectomy (0.98 ± 0.6 mmol/L vs. 0.58 ± 0.6 mmol/L; *P* < 0.01).

### Effect of open splenectomy on the activities of AST and ALT, and concentration of urea and creatinine

**Figure 8:**
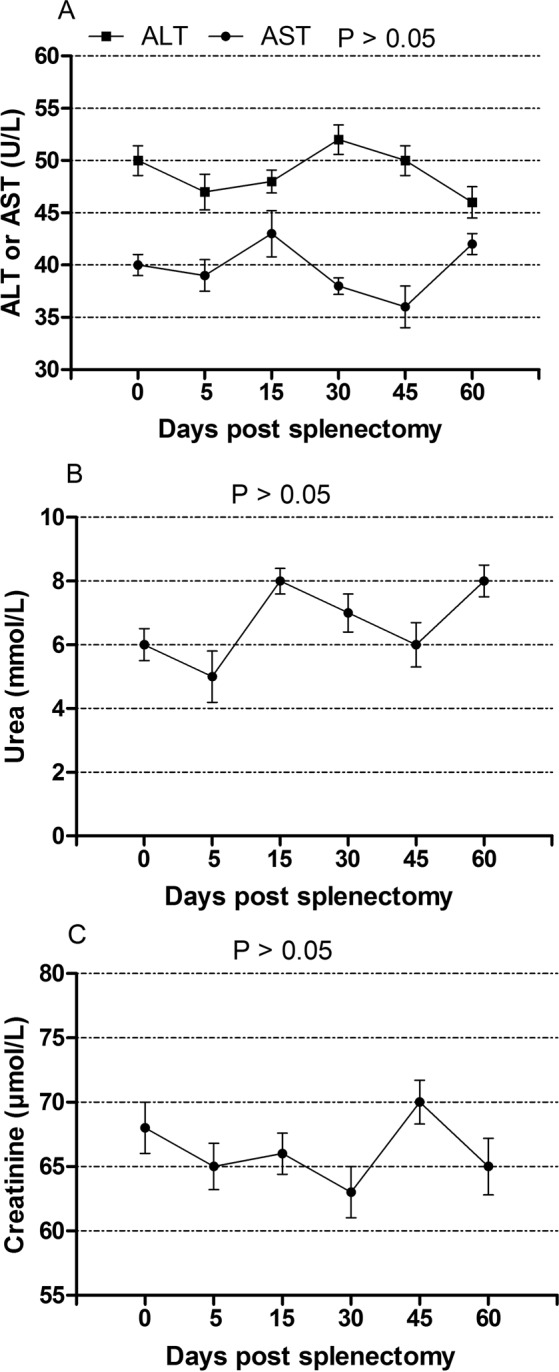
The values (mean ± SE) of liver and kidney function indices in dogs after splenectomy. (**A**) AST and ALT activities. (**B**) Urea. (**C**) Creatinine.

The effect of splenectomy on serum AST, ALT, urea and creatinine is displayed in Fig. 8. No significant variations in the tested parameters were noticed during the study period (*P* > 0.05).

## Discussion

In dogs, open splenectomy is mandatory as an emergency following splenic rupture with resultant hemoperitoneum and hypotensive shock^18^, as well as it may be indicated in canine with diffuse (hemangiosarcoma, splenitis, amyloidosis) or nodular (hematoma, abscess, hemangioma) splenomegaly^6^. In the current study, to avoid the large blood vessels in the subcutis and muscle^15^, the abdomen was opened along its ventral midline. In human medicine, thromboembolic complications in the form of DVT and consequent thromboembolism may occur in up to 10% of patients following splenectomy^19^. As reported in previous studies^20,21^, Doppler ultrasonography has emerged as the preferred technique for the diagnosis of DVT. In the current study, Doppler ultrasonographic examination was carried out on brachial and femoral veins because these veins are deeply located, as well as they are suggested as common sites for the development of DVT in human patients^22,23^. In the present work, Doppler ultrasonography revealed no evidence of DVT. In contrary to this finding, Rodeghiero and Ruggeri^24^ suggested the development of DVT in patients as a long-term complication after splenectomy. Such variation could be attributed the short-term course of the present study.

In comparison with the baseline values, the mean WBCs counts were significantly higher on day 8 after surgery. Such leukocytosis may be due to transient non-specific infection as a consequent of splenectomy and/or the stress of operation. As reported elsewhere^18^, the spleen is a member of the reticuloendothelial system that contains local macrophages responsible for phagocytosis and lymphoid tissues involved in immunosurveillance and the production of B and T lymphocytes. Furthermore, increased corticosteroid caused by fear or other stresses of anesthesia or laparotomy may also be associated with a moderate increase in WBCs^25^. However, in the present study, all dogs were apparently clinically healthy throughout the study period; therefore, increased WBCs could be a normal physiological response to stress. Such leukocytosis had been recorded in human patients following splenectomy and was attributed to the physiological changes^26^.

After splenectomy, dogs exhibited a drop in RBCs, Hb, and HCT, indicating anemia. Such decrease in erythrogram indices could be attributed to the loss of red blood cells reserve in the spleen after the operation. As listed in a previous study^18^, the spleen is essential for extramedullary erythropoiesis and acts as a reservoir for about 20% of erythrocytes. Another explanation for such drop was described in a recent study^27^, the authors attributed the anemic state after splenectomy to the reduction of serum iron concentrations.

In the present study, the coagulation profile revealed persistently increased PLT counts after splenectomy and throughout the entire postoperative period. This finding was supported in previous studies in human patients after splenectomy^26,28,29^. As discussed before^30^, this increase may occur as a result of secondary response for splenectomy. It had been indicated that increased thrombopoietin and interleukin-1 in patients are the cause of thrombocytosis following splenectomy^31^. In a recent study^32^, the authors reported reactive thrombocytosis with a prevalence of up to 75% after splenectomy.

However Doppler ultrasonography, in the present study, showed no evidence of venous thrombosis and/or DVT, thrombocytosis may act as a potential risk for thrombus formation. Supporting this presumption is that in a previous research work^33^, the authors reported thromboembolic complications and DVT in 10% of human patients following splenectomy. Crary and Buchanan^2^ stated that loss of the spleen’s filtering activities may allow damaged cells to persist in the circulation, leading to changes in the endothelium that result in hypercoagulability. In addition, other changes that have been reported to occur after splenectomy that might potentially contribute to thrombosis risk include increased platelet and leucocyte counts^19^.

In the current study, PT and APTT showed no significant variations throughout the entire postoperative period, indicating no direct influence of splenectomy on the coagulation times. As mentioned elsewhere^34^, PT is controlled by the extrinsic pathway factors and APTT is controlled by the intrinsic pathway factors. Since both extrinsic and intrinsic factors are produced from the liver^35^, and the liver enzyme activities in the present research were relatively stable, this explains why PT and APTT values showed no great changes. In contrast, a recent study^36^ concluded improvement of hemostatic and liver function in hepatosplenic *schistosomiasis mansoni* infected patients following splenectomy. Such difference could be attributed to the disease involvement of liver and spleen in that research.

After splenectomy, the mean serum concentrations of total cholesterol, LDL and TG were significantly increased, indicating alteration of lipid profile. Such variation could be attributed to the role of the spleen in lipid metabolism. In a previous study, the authors proposed the presence of a “splenic factor” responsible for lipid metabolism^37^. However, the accurate mechanisms behind this mysterious phenomenon remain unclear so far^38^. In a recent research work on rats, the authors reported increased cholesterol, LDL and TG, and decreased HDL concentrations after complete splenectomy, then these values reverted by autogenous spleen tissue implants^4^. In the present study, increased lipid parameters may have a potentiality for development of atherosclerosis and consequently vascular complications. Experimentally, many studies have provided evidence of increased risk and promotion of atherosclerotic changes in the absence of spleen^39,40^.

In comparison with their baseline values, the activities of AST and ALT, and concentrations of urea and creatinine showed no significant changes following the surgical operation, indicating no harmful effect of splenectomy on liver and kidney functions. This postulation was supported by another study, which had reported improvement of liver function in patients with chronic viral hepatitis C following splenectomy^41^.

As the course of the present research was relatively short, Doppler ultrasonographic evaluation of brachial and femoral veins revealed no evidence for venous thrombosis or other vascular complications. Moreover, laboratory indices revealed leukocytosis, thrombocytosis, and increased total cholesterol, LDL and TG, which may act as predisposing factors for the development of vascular complications with the long run. In a recent review^37^, the authors mentioned that increased platelets and WBCs, and increased lipid variables have the risk for vascular thrombosis.

## Conclusion

Open splenectomy may have no influence on the Doppler ultrasonographic parameters of brachial and femoral veins, and no evidence for DVT in dogs. Furthermore, persistent anemia, leukocytosis, and thrombocytosis, as well as alteration of lipid profile may develop following splenectomy. Therefore, the risk of vascular complications may still exist. However, a further long-term study may be necessary.
